# Evaluating the Safety of Immune Checkpoint Inhibitors and Combination Therapies in the Management of Brain Metastases: A Comprehensive Review

**DOI:** 10.3390/cancers16233929

**Published:** 2024-11-23

**Authors:** Vivek Podder, Tulika Ranjan, Kim Margolin, Arun Maharaj, Manmeet Singh Ahluwalia

**Affiliations:** 1 Miami Cancer Institute, Baptist Health South Florida, Miami, FL 33186, USA; drvivekpodder@gmail.com (V.P.); dr.ranjan2017@gmail.com (T.R.); arun.maharaj@baptisthealth.net (A.M.); 2 Saint John’s Cancer Institute, Santa Monica, CA 90404, USA; kim.margolin@providence.org

**Keywords:** brain metastasis, non-small-cell lung cancer, melanoma, immune checkpoint inhibitors, immune-related adverse events, stereotactic radiosurgery

## Abstract

Brain metastases (BM) are a frequent and severe complication in patients with lung cancer, breast cancer, and melanoma. Immune checkpoint inhibitors (ICIs) have become a crucial treatment option for BM, whether used alone or in combination with chemotherapy and stereotactic radiosurgery (SRS). However, ICIs are associated with immune-related adverse events (irAEs) that can affect multiple organ systems, complicating their use in BM patients. This review examines the mechanisms of irAEs and their effects on different organs and evaluates the safety of ICIs across various treatment strategies for BM. Our analysis indicates that ICIs significantly improve survival and disease control in BM patients, but their use increases the risk of irAEs, including dermatologic, gastrointestinal, endocrine, pulmonary, and neurologic toxicities. Neurotoxic events, particularly treatment-associated brain necrosis (TABN) and encephalitis, are more common in BM patients. While the overall incidence of irAEs is similar between patients with and without BM, the neurotoxicity risk is higher in the BM population. Combining ICIs with chemotherapy and SRS enhances efficacy but also heightens the risk of adverse events across organ systems. ICIs offer substantial benefits for BM patients but require careful management to mitigate the risks of irAEs. Close patient monitoring, individualized treatment protocols, and prompt intervention are essential for optimizing the outcomes. Future research should focus on refining combination strategies and improving the management of irAEs, particularly neurotoxicity, to maximize therapeutic benefits for BM patients.

## Simple Summary

Immune checkpoint inhibitors (ICIs) have become an important treatment for patients with brain metastases from cancers like lung cancer, melanoma, and breast cancer. While ICIs can improve survival, they can also cause immune-related adverse events (irAEs), affecting various organs, including the brain. This review discusses the safety of ICIs when used alone or in combination with other treatments like chemotherapy and radiosurgery. We explain how irAEs occur, their effects on different parts of the body, and how to manage them. Careful monitoring and treatment planning are essential to ensure the best outcomes for patients with brain metastases.

## 1. Introduction

Brain metastases (BM) are the most common malignant brain tumors, primarily arising from lung cancer, breast cancer, and melanoma [1]. In the United States, BM affects approximately 200,000 individuals annually, accounting for 10–30% of all cancer cases [1,2,3], and can cause significant morbidity and mortality [4,5]. Local brain-directed therapies such as stereotactic radiosurgery (SRS), whole-brain radiation therapy (WBRT), and neurosurgical resection are standard treatment options for managing BM. However, these treatments are often insufficient due to tumor heterogeneity and the complex tumor microenvironment (TME).

Immune checkpoint inhibitors (ICIs) have demonstrated promise in treating BMs, whether as monotherapy or combined with other therapies. However, ICIs are associated with immune-related adverse events (irAEs), which can be acute or chronic and vary widely in severity and the organs they affect [6]. The rising use of ICIs has increased the incidence of irAEs, making it essential for oncologists to understand their mechanisms and effects across organ systems. Combining ICIs with chemotherapy and SRS has shown potential for enhanced efficacy in BM treatment, but it increases the risk of neurotoxicity and chronic irAEs post-treatment, adversely affecting the quality of life [7,8]. Neurotoxicity, particularly when ICIs are combined with SRS, remains a major concern due to evidence of worsened neurologic irAEs [9,10].

Although ongoing clinical trials are investigating the safety and efficacy of combining ICIs with chemotherapy and SRS, comprehensive data are still pending and are of critical need [10]. This review aims to examine the mechanisms underlying irAEs, categorize their general effects on different organ systems, and critically assess the safety of ICIs as monotherapy or combined with chemotherapy and SRS in treating BM.

## 2. Mechanisms of irAEs Associated with Immune Checkpoint Inhibitors

ICI-related irAEs result from an imbalance between immune activation and tolerance, driven by autoreactive T-cells, B-cells, and cytokines such as IL-6, IL-8, and TNF [11]. This immune imbalance, influenced by the TME, causes tissue damage in organs reliant on T-cell tolerance (skin and colon) [11,12]. Regulatory T-cell (Treg) dysfunction and elevated CD4 effector memory T-cell levels contribute to irAE severity, while self-reactive B-cells and auto-antibodies exacerbate tissue damage [11,12]. Inflammatory cytokines amplify immune damage, while disturbances in gut microbiota can trigger irAEs [11].

The risk and severity of irAEs differ across ICIs. CTLA-4 inhibitors generally cause higher irAE rates, such as hypophysitis and colitis, whereas PD-1/PD-L1 inhibitors are more often linked to pneumonitis and myocarditis [13]. Nivolumab and ipilimumab combination therapy increases toxicity compared to monotherapy, demonstrating a synergistic effect [13]. Additionally, combining ICIs with treatments targeting EGFR and ALK pathways, especially in advanced NSCLC, heightens the risk of severe irAEs like pneumonitis and hepatitis [13]. While these effects resemble autoimmune diseases, the underlying mechanisms remain unclear. Understanding the mechanisms of irAEs is critical for managing the wide range of organ-specific irAEs that can emerge during treatment.

## 3. Organ-Specific Immune-Related Adverse Events (irAEs)

**Figure 1:**
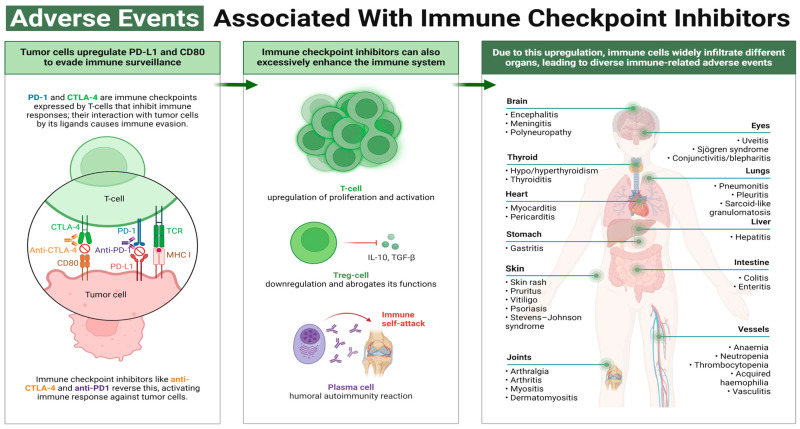
Adverse events associated with immune checkpoint inhibitors (ICIs). The upregulation of immune checkpoints PD-1 and CTLA-4 on T-cells inhibit immune responses, causing immune invasion. ICIs like anti-CTLA-4 and anti-PD-1 reverse this process and activate an immune response against tumor cells (*left panel*). However, although ICIs enhance the immune system via increased T-cell proliferation and activation, the inhibition of cytokine release (IL-10 and TGF-β) via regulatory T-cell (Treg) downregulation increases the humoral autoimmune reaction (*middle panel*). As a result, the upregulation of immunity causes immune cells to infiltrate various organs, leading to many immune-related adverse events (*right panel*). Created with BioRender.com.

By activating T-cells, ICIs cause a wide range of irAEs across organ systems, posing challenges for clinicians due to their varying onset, severity, and frequency (Figure 1). While irAEs often occur within the first 3 months of treatment, they can also emerge long after discontinuation. Although many irAEs resolve, some persist as chronic conditions, requiring ongoing management with hormonal supplements or immunosuppressive therapies.

### 3.1. Dermatologic Immune-Related Adverse Events (irAEs)

Dermatologic irAEs affect 33–50% of patients undergoing ICI therapy [14,15]. Anti-CTLA-4 and anti-PD-1 therapies frequently cause rashes, pruritus, and vitiligo, which is linked to improved tumor responses in melanoma [15]. Most dermatologic irAEs, such as maculopapular rashes covering <30% of the body surface area (BSA), resolve within 1–2 months, though some patients experience mild recurrent skin toxicities [16,17,18]. Severe skin irAEs (≥30% BSA) occur in 2–3% of ICI monotherapy cases and 4–10% of combination therapy cases, presenting as exfoliative or bullous dermatitis [19]. Less common dermatologic irAEs include dry skin, mouth sores, hives, hair changes, and conditions resembling dermatomyositis and lupus-like dermatoses [14]. Skin toxicities are more diverse and often develop later with PD-1/PD-L1 inhibitors, while combination ICI therapies typically lead to earlier and more severe reactions [19,20,21].

### 3.2. Lower Gastrointestinal Tract Immune-Related Adverse Events (irAEs)

Diarrhea and colitis are the most common gastrointestinal irAEs in ICI therapy, with colitis rates of 10–20% under ipilimumab treatment [22]. Endoscopy often reveals widespread ulceration and edema in the colon, and around 25% of patients also experience diffuse enteritis independent of colitis [23]. Despite acute presentation, the epithelial architecture remains intact (unlike in inflammatory bowel disease), and biopsies may reveal microscopic colitis without visible disease [24]. Early treatment of high-grade colitis with infliximab and corticosteroids has shown faster symptom resolution and reduced steroid use, supporting early utilization of biological agents to prevent chronic inflammation.

CTLA-4 inhibition disrupts mucosal self-tolerance by depleting regulatory T-cells in the gut, and genetic factors like CTLA-4 polymorphisms may increase the risk of early-onset Crohn’s disease [25,26]. Specific gut bacterial strains protect against anti-CTLA-4 colitis in mice, and in humans, a high abundance of Bacteroides is linked to a lower incidence of colitis [27,28,29]. Fecal microbiota transplantation has effectively treated steroid- and anti-TNF refractory colitis, altering gut-immune infiltrates [30]. The pathophysiology of anti-PD-1-induced colitis differs from that of ipilimumab. Anti-PD-1 therapy is associated with less frequent colitis or enteritis, suggesting that the PD-1 pathway may play a lesser role in maintaining the gut immune balance [31,32].

### 3.3. Pulmonary Immune-Related Adverse Events (irAEs)

Pneumonitis occurs in approximately 5% of patients receiving ICI therapy, can be life-threatening [33,34,35,36], and is less common in monotherapy with anti-PD-1/PD-L1 antibodies (3%) compared to combination therapy with anti-CTLA-4 antibodies (10%) [35]. The median onset is earlier in combination therapy (2.7 months) than in monotherapy (4.6 months), though delayed cases can appear >1 year after treatment initiation [35,37]. Diagnosing pneumonitis is particularly challenging in lung cancer patients due to pre-existing lung conditions, with cryptogenic organizing pneumonia being the most common radiographic finding [35,38,39,40].

Pneumonitis is more common and severe in NSCLC than in melanoma patients, particularly with first-line anti-PD-1/PD-L1 therapies [33,41]. Factors such as prior chemotherapy, radiation, lung disease, and smoking history contribute to the risk and severity of pneumonitis, with lower rates observed when ICI therapy is used as a second-line treatment.

### 3.4. Endocrine Immune-Related Adverse Events (irAEs)

Endocrine irAEs are common complications in patients receiving ICIs, with a 10% incidence of clinically significant cases reported across 38 trials [42,43,44]. These irAEs most commonly involve conditions such as hypothyroidism, hyperthyroidism, and hypophysitis. Less frequent but notable are type-1 diabetes (0.2–0.9%) and adrenal insufficiency (0.7%) [44,45]. This subsection provides an overview of the different endocrine irAEs, including their mechanisms, clinical presentation, and strategies for diagnosis and treatment.

#### 3.4.1. Hypophysitis

Hypophysitis is particularly prevalent among patients treated with ipilimumab, with real-world incidence rates of 12.0–13.3% [45,46]. The diagnosis is based on clinical symptoms, hormone imbalances, and MRI findings, though early scans may appear normal [47]. In a multicenter study, pituitary enlargement was common in patients with ipilimumab-induced hypophysitis, typically resolving within 6 weeks, although central adrenal insufficiency often persists [48,49,50].

Iwama et al. identified that ipilimumab-induced hypophysitis is likely driven by the direct binding of anti-CTLA-4 antibodies to CTLA-4-expressing pituitary cells, leading to antibody-dependent cell-mediated cytotoxicity (ADCC) and complement-mediated cytotoxicity (CDC) [51]. Caturegli et al. confirmed CTLA-4 expression in nonmalignant and adenomatous pituitary cells, suggesting a type-II hypersensitivity reaction involving CDC [52]. Tremelimumab, an IgG2 anti-CTLA-4 antibody, has shown a lower incidence of hypophysitis due to reduced complement activation potential [53]. However, ADCC may still lead to hypophysitis, as demonstrated by a case of severe hypophysitis in a patient with elevated pituitary CTLA-4 levels treated with tremelimumab [54]. Rare cases of hypophysitis have also been observed with anti-PD-1 therapies, suggesting that multiple mechanisms—ADCC, CDC, and direct cell-mediated cytotoxicity—contribute to ICI-induced hypophysitis.

#### 3.4.2. Thyroid Immune-Related Adverse Events (irAEs)

Thyroid irAEs vary between anti-PD-1 and anti-CTLA-4 therapies and are exclusively linked to anti-PD-L1 antibodies [55]. Approximately 20% of patients on anti-PD-1 therapy develop thyroid disease roughly 6 weeks after treatment initiation [56,57]. Hypothyroidism rates range from 3.8% to 13.2%, and hyperthyroidism from 0.6% to 8% across treatments like ipilimumab, nivolumab, pembrolizumab, and atezolizumab [43].

Most thyroid irAEs are mild and asymptomatic, presenting as transient thyrotoxicosis or hypothyroidism, often due to thyroiditis [57,58]. In a study of melanoma patients on pembrolizumab, many cases transitioned from hyper- to hypothyroidism within 1–3 months [56]. Positive anti-thyroglobulin antibodies and increased fluorodeoxyglucose (FDG) uptake on PET/CT scans may predict subsequent hypothyroidism [59,60]. A cohort study of 1781 patients showed that those who developed thyroid dysfunction had significantly improved overall survival compared to those without thyroid irAEs (41 vs. 22 months) [61].

The treatment of ICI-induced thyrotoxicosis is mainly supportive, as it is usually short-lived and asymptomatic, often preceding hypothyroidism. High-dose glucocorticoids did not alter the course of thyrotoxicosis or progression to hypothyroidism in a study of 53 patients, and beta-blockers are used for symptom relief [62]. Persistent hypothyroidism requires levothyroxine treatment after ruling out adrenal insufficiency. The pathophysiology of ICI-induced thyroid dysfunction remains unclear, with variable recovery rates ranging from complete recovery to persistent hypothyroidism [57,63].

### 3.5. Hepatic Immune-Related Adverse Events (irAEs)

Hepatic toxicity is a notable irAE in ICI therapy, particularly in patients treated with CTLA-4 and PD-1/PD-L1 inhibitors, either alone or in combination. ALT and AST elevations occur in 3–9% of those patients on CTLA-4 inhibitors, 1.5–5% for PD-1 inhibitors, and 3% for PD-L1 inhibitors, with severe hepatotoxicity being rare, at around 1% [64,65,66,67,68]. However, the combination of CTLA-4 and PD-1 inhibitors increases the risk, with 15–20% of patients experiencing enzyme elevations and 4–9% developing severe (≥grade-3) liver injury [65,69,70,71].

ICI-induced hepatitis typically manifests as isolated transaminase elevations, often resolving after treatment discontinuation, but severe cases with liver dysfunction, including hyperbilirubinemia and coagulopathy, have been reported [72,73]. The guidelines recommend suspending ICIs at grade-2 liver transaminase elevations (2–5 times the upper limit) and discontinuing at higher levels. Liver biopsies are advised for moderate-to-severe enzyme elevations to exclude other causes [72]. A phase-I trial in renal cell carcinoma showed that ipilimumab 3 mg/kg combined with nivolumab 1 mg/kg resulted in higher hepatotoxicity (20% grade 3–4 hepatitis) compared to the reverse dosing regimen (6.4%) [74].

### 3.6. Cardiac Immune-Related Adverse Events (irAEs)

Cardiac irAEs associated with ICIs include myocarditis, pericarditis, heart failure, and arrhythmias, with myocarditis being particularly concerning due to its fatal potential and variable onset. The risk of myocarditis is higher with combination immunotherapy than single-agent therapy [75,76]. A pharmacovigilance study found 18 severe myocarditis cases among 20,594 patients, resulting in an incidence of 0.09%, with a higher rate in combination therapy (0.27%) compared to nivolumab alone (0.06%) [75]. The mortality rate was also higher in the combination group (67%) versus the monotherapy group (36%) [77]. Mechanistically, ICI-induced myocarditis is believed to result from a breakdown in self-tolerance driven by a T-cell-mediated inflammatory response. Autopsies revealed myocardial infiltration by CD8+ T-cells, and studies identified identical T-cell receptors in tumor and myocardial tissue, indicating a T-cell-mediated attack rather than an antibody-driven process [78].

Due to the high fatality risk, early and aggressive management is crucial. Initial treatment involves high-dose corticosteroids (prednisone 1–2 mg/kg/day) [75,76,77]. If there is no immediate response, escalation to methylprednisolone (1g/day) and adding immunosuppressive agents like mycophenolate, infliximab, or antithymocyte globulin is necessary. Patients with elevated troponin or conduction abnormalities require transfer to a coronary care unit. In life-threatening cases, abatacept or alemtuzumab is used.

### 3.7. Neurologic Immune-Related Adverse Events (irAEs)

Neurologic irAEs from ICI therapy affect the central, peripheral, and autonomic nervous systems, with prevalence ranging from 1% to 14%, notably higher in patients receiving combined nivolumab and ipilimumab [79,80,81]. Severe manifestations include limbic encephalitis, aseptic meningitis, Guillain–Barré syndrome (GBS), transverse myelitis, myasthenia gravis (MG), and inflammatory myopathies. MG and encephalitis are more commonly associated with anti-PD-1 therapies, while meningitis is often seen with anti-CTLA-4 therapies [79,80]. A review of 9000 patients reported neurologic irAEs in 4% of those on CTLA-4 blockers, 6% of those on PD-1 inhibitors, and 12% of those on combined therapy [82].

Mechanistically, ICI-related irAEs result from disruptions in immune tolerance, leading to an overactive immune response where autoreactive T-cells, B-cells, and cytokines such as IL-6, IL-8, and TNF contribute to immune-mediated damage in neurological tissues. Animal studies further elucidated this; for example, mouse models showed cerebellar inflammation and T-cell infiltration in 84% of mice treated with anti-CTLA-4 antibodies, expressing a neo-self-antigen in Purkinje and tumor cells, compared to no inflammation in controls, implicating T-cell dysregulation in neurologic toxicities [83]. Encephalitis is typically T-cell-mediated, with cerebrospinal fluid autoimmune and paraneoplastic panels often negative, suggesting undetectable autoimmune antibodies [84]. ICI therapy-induced encephalitis often manifests as a T-cell-mediated process, like other irAEs, rather than stemming from paraneoplastic syndromes. Cerebrospinal fluid paraneoplastic and autoimmune antibody panels are frequently negative, indicating the importance of clinical diagnosis and the potential existence of undetectable autoimmune antibodies [84]. Acetylcholine receptor antibodies, found in 85% of generalized MG patients, are frequently absent in ICI-associated MG, with up to 33% of these cases also reporting myositis and 8% presenting concurrent myocarditis [85,86,87]. Peripheral neuropathies associated with ICIs include pain, sensory or motor neuropathies, cranial neuropathies, and inflammatory polyradiculopathies like GBS [81,88].

Neurologic irAEs require prompt intervention due to the potential for rapid progression. For MG, ICIs are discontinued, and corticosteroids (prednisone 0.5 mg/kg/day) and pyridostigmine are administered. In severe cases, intravenous immunoglobulin (IVIG) treatment or plasmapheresis is initiated. GBS is managed with IVIG treatment or plasmapheresis, and corticosteroids may be used in ICI-related cases. Encephalitis is treated with corticosteroids, escalating to IV pulse steroids in more severe cases, with IVIG treatment or plasmapheresis as necessary.

### 3.8. Systemic Adverse Events Associated with Immune Checkpoint Inhibitors (ICIs)

ICIs are linked to systemic adverse events, including fatigue, infusion-related reactions, and cytokine release syndrome (CRS). Fatigue affects 16–24% of patients on PD-1/PD-L1 inhibitors and 26% of patients on combination therapies, though it is generally mild and requires ruling out other causes, such as endocrine disorders [89,90]. Infusion-related reactions, seen in up to 25% of cases (especially with avelumab), are typically mild and managed with acetaminophen and antihistamines, while severe reactions may necessitate stopping the treatment. CRS, an acute inflammatory response associated with CAR-T therapy, has also been observed with ICIs like nivolumab, potentially causing fever and multi-organ dysfunction. Effective management strategies are essential to minimize these systemic effects while optimizing the benefits of immunotherapy [91].

## 4. Comparative Analysis of irAEs in Single-Agent and Dual-Agent ICI Therapy from Clinical Trials in Brain Metastasis (BM)

### 4.1. Single-Agent and Dual-Agent ICI Therapy for NSCLC with Brain Metastases

ICIs such as pembrolizumab are widely used as monotherapy for NSCLC patients with high PD-L1 expression, providing a frontline treatment option without chemotherapy. Nivolumab and atezolizumab are also approved for advanced NSCLC after chemotherapy. Managing irAEs is particularly critical in BM, as these toxicities can involve multiple organs, including the brain. In a phase-II trial of pembrolizumab for NSCLC patients with untreated BMs, grade-3 irAEs such as pneumonitis (5%), colitis (2%), adrenal insufficiency (2%), and hyperglycemia (2%) were observed [92]. In an expanded access program evaluating nivolumab in NSCLC patients with BM, 7% of patients experienced irAEs, with grade 3–4 irAEs including pneumonitis, elevated transaminase levels, and hypothyroidism, all managed with protocol-defined strategies [93]. Fatal irAEs, although rare, have been reported in NSCLC patients receiving ICIs, with myocarditis and pneumonitis being particularly concerning. While systemic irAEs are similar to those seen in NSCLC patients without BM, the presence of BM increases the risk of neurological complications such as encephalopathy and seizures. Close monitoring of the neurologic function, early detection, and prompt management of irAEs are essential to ensure patient safety during treatment. Table 1 summarizes immune-related adverse events (irAEs) from key trials on ICIs and combination therapies in BM patients.

### 4.2. Single-Agent and Dual-Agent ICI Therapy for Melanoma with Brain Metastases

Melanoma BM (MBM) presents unique challenges, particularly in patients without BRAF mutations, where ICIs have demonstrated significant efficacy. However, managing irAEs remains a critical concern, as observed in NSCLC with BM. Dual ICI therapy offers a higher iORR but also carries more significant toxicity. Nivolumab, combined with ipilimumab, is highly effective but carries a higher risk of toxicity. In the CheckMate 204 trial, 55% of asymptomatic MBM patients experienced grade 3–4 irAEs, primarily liver enzyme elevations [94]. Serious irAEs included colitis, hypophysitis, and increased alanine aminotransferase, occurring in about 5% of patients, with one death from myocarditis. In the ABC trial, 60 asymptomatic patients with untreated BM were treated with nivolumab, ipilimumab, or both [95]. No new long-term toxicities were reported. Single-agent ICIs like ipilimumab, pembrolizumab, and nivolumab offer safer profiles with fewer severe irAEs. They remain effective in treating melanoma BM, making them suitable for patients intolerant to combination therapies. In the phase-II trial of pembrolizumab for active MBM, most irAEs were mild (grades 1–2) [96]. Grade-3 irAEs included hepatitis and rash, while neurologic irAEs, such as seizures and perilesional edema, were also observed but were generally manageable with corticosteroids and antiepileptics. No treatment-related deaths occurred. While dual therapy increases the risk of severe irAEs, including neurologic events, single-agent ICIs offer a safer alternative, particularly for patients who are not candidates for combination therapy.

### 4.3. Single-Agent and Dual-Agent ICI Therapy for Triple-Negative Breast Cancer (TNBC) with Brain Metastases

Data on ICI use in triple-negative breast cancer (TNBC) with BM is limited, with most trials including only small subsets of BM patients. Specific data on irAEs in this subgroup is lacking. This lack of data may be due to the historically poor prognosis of TNBC patients with brain BM who were excluded from clinical trials. TNBC with BM may exhibit different biological behaviors than other subtypes, requiring further exploration. Future studies focusing on evaluating the unique tumor biology of TNBC with BM and identifying biomarkers that predict response to ICI therapies in this subgroup are of critical need.

## 5. Comparative Analysis of irAEs in ICI and Chemotherapy Combination Therapy from Clinical Trials in Brain Metastasis (BM)

### 5.1. ICI and Chemotherapy Combination Therapy for NSCLC with Brain Metastases

In addition to their use as monotherapy, ICIs are increasingly combined with chemotherapy to enhance therapeutic efficacy, particularly in treating BMs, especially in NSCLC without driver mutations. Patients with asymptomatic BM often undergo chemoimmunotherapy, which, though effective, carries an increased risk of irAEs. Clinicians select these regimens based on molecular markers, such as PD-L1 expression, patient performance status, and prior treatment history. In the Atezo-Brain phase-II trial, atezolizumab combined with carboplatin and pemetrexed showed grade 3–4 irAEs in 27.5% of patients, including pneumonitis and acute kidney injury (5%) [97]. Building on these findings, the pooled analysis of the KEYNOTE-021, −189, and −407 trials found that 25.5% of NSCLC patients with BM had ≥grade-3 irAEs, including pneumonitis and nephritis [98]. Neurologic events, such as encephalopathy, occurred in 32.4% of patients, contributing to a 5.9% rate of treatment-related deaths. The safety profile in patients with BM was consistent with those without BM.

In KEYNOTE-189, an updated analysis confirmed the safety of pembrolizumab combined with chemotherapy [99]. A total of 10.9% of patients experienced grade 3–4 irAEs, including pneumonitis (3%), colitis (1.5%), and nephritis (1.5%) [94]. The safety profile was consistent between patients with and without BM, underscoring the feasibility of pembrolizumab-based regimens in this subgroup without added toxicity. Similarly, in the CheckMate 9LA trial, nivolumab plus ipilimumab combined with chemotherapy showed grade 3–4 irAEs in 43% of patients with BM, including rash (18%) and hepatitis (4%) [100].

Combining ICIs with chemotherapy in NSCLC patients with BM is effective but increases the risk of higher-grade irAEs, particularly pneumonitis and encephalopathy, which can be fatal. While the overall safety profile in BM patients is similar to that of patients without BM, neurologic irAEs require particular attention due to the elevated risk of encephalopathy and its contribution to treatment-related mortality.

### 5.2. ICI and Chemotherapy Combination Therapy for SCLC with Brain Metastases

In extensive-stage small-cell lung cancer (ES-SCLC), ICIs are part of frontline therapy. In the IMpower133 trial, atezolizumab added to carboplatin and etoposide in ES-SCLC showed a comparable safety profile between patients with and without BM [101]. The study found that irAEs, such as rash and hypothyroidism, were more frequent in the atezolizumab group (39.9%) compared to placebo (24.5%) but remained manageable. In the CASPIAN trial, which evaluated durvalumab combined with platinum–etoposide in the frontline treatment of ES-SCLC with and without BM, grade 3–4 irAEs occurred in 5% of patients in the combination group [102]. The most common irAEs, including hypothyroidism and hyperthyroidism, were generally low-grade and manageable. One case of hepatotoxicity was reported, suggesting that durvalumab plus chemotherapy may be a safer option for BM patients compared to chemotherapy alone.

### 5.3. ICI and Chemotherapy Combination Therapy for Triple-Negative Breast Cancer (TNBC) with Brain Metastases

Managing TNBC with BM is challenging, and ICIs combined with chemotherapy offer new treatment possibilities. The KEYNOTE-355 trial evaluated pembrolizumab plus chemotherapy in metastatic TNBC, including 3% of patients with treated and stable BM [103]. irAEs occurred in 26% of patients receiving pembrolizumab compared to placebo (6%). Common irAEs included hypothyroidism (15%), hyperthyroidism (5%), pneumonitis (2%), colitis (2%), and severe skin reactions, with 5% experiencing ≥grade-3 events. No irAE-related deaths were reported. In the IMpassion130 trial, atezolizumab with nab-paclitaxel caused grade 3–4 irAEs in 8% of patients, including neutropenia (8%) and peripheral neuropathy (6%) [104]. One case of autoimmune hepatitis leading to death in the atezolizumab group was reported. In the ENHANCE 1 phase Ib/II trial, eribulin plus pembrolizumab in metastatic TNBC, including patients with stable BM, resulted in grade 3–4 irAEs in 12% of patients, including hypothyroidism (18%) and pneumonitis (11%) [105]. No treatment-related deaths were reported. In TNBC patients with BM, ICIs combined with chemotherapy have demonstrated a manageable safety profile, with most irAEs being low-grade. Although grade 3–4 events such as pneumonitis and hypothyroidism occur, treatment-related deaths are rare.

In TNBC patients with BM, ICIs combined with chemotherapy have demonstrated a manageable safety profile, with most irAEs being low-grade. However, fatal irAEs, such as autoimmune hepatitis, have been reported in rare cases.

### 5.4. ICI and Chemotherapy Combination Therapy for Melanoma with Brain Metastases

Cytotoxic chemotherapy has limited efficacy in MBM. However, combining ICIs with chemotherapy has shown promise despite safety concerns. In the NIBIT-M2 phase-III trial, patients treated with ipilimumab plus nivolumab experienced grade 3–4 irAEs in 30% of cases, primarily liver (43%) and skin-related (37%) [106]. In the ipilimumab plus fotemustine arm, 38% of patients had grade 3–4 irAEs, though no unexpected toxicities or deaths were reported.

## 6. Comparative Analysis of irAEs in ICI and Stereotactic Radiosurgery (SRS) Combination Therapy in Clinical Trials for Brain Metastasis (BM)

Alongside chemotherapy, combining ICIs with SRS has emerged as another promising approach, although it raises concerns regarding neurotoxicity. The timing and sequencing of these therapies play a crucial role in influencing safety outcomes, and existing evidence presents a mixed picture. SRS is effective for BM due to its precision and fewer neurocognitive side effects than WBRT. Combining SRS with ICIs may increase the risk of neurotoxicity, particularly treatment-associated brain necrosis (TABN), which typically emerges around 11 months post-treatment, with incidence rates ranging from 5.9% to 17.5% [107].

Martine et al. reported a significant increase in symptomatic TABN, with 20% of patients treated with SRS plus ICIs developing this condition, compared to 7% of those receiving SRS alone [108]. Similarly, Kaidar et al. found that 28% of patients with MBM treated with the combination developed TABN, with hemorrhages occurring in 25% of these patients, compared to only 6.89% in the SRS-alone group [109]. However, these findings are contrasted by studies like those of Colaco et al. and Patel et al., which indicate that the risk of TABN might not be significantly higher when combining SRS with ICIs. Colaco et al. reported a 37.5% incidence of TABN in those patients receiving ICIs, compared to 25% in patients with targeted therapy and 16.9% in patients with chemotherapy. Patel et al. reported similar TABN rates between those patients treated with SRS alone (21%) and those receiving SRS plus ICIs (30%), suggesting that the risk may vary based on tumor histology and treatment timing [110,111].

The timing of ICI administration relative to SRS is particularly critical. Chen et al. found no significant increase in CNS toxicity or irAEs when SRS and ICI were administered concurrently (within 2 weeks), with CNS toxicity rates of 30% in the concurrent group versus 32% in the non-concurrent group [112]. Kotecha et al. also reported similar 12-month cumulative rates of radiation necrosis—3.2% in patients treated with immediate ICI (±1 half-life of the ICI) versus 3.5% in all patients treated with concurrent or non-concurrent SRS plus ICI [113]. However, studies like that of Koenig et al. showed that concurrent administration (within 4 weeks) led to a higher risk of adverse radiation events, with a hazard ratio of 4.47 for increased radiation necrosis compared to non-concurrent administration [114]. Kiess et al. also reported an increased risk of grade 3–4 adverse events in the concurrent group, emphasizing the potential risks associated with timing [115]. While combining SRS with ICIs offers improved survival and local control for patients with BM, the timing of administration is critical in managing the risk of neurotoxicity. The evidence is mixed, with some studies showing increased risks with concurrent administration while others report no significant difference. These findings highlight the need for large-scale, long-term, and randomized controlled trials to clarify SRS and ICI therapy’s optimal timing and sequencing to ensure safety and efficacy.

## 7. Conclusions and Future Directions

ICIs provide a promising treatment option for BM, but irAEs pose significant risks that need careful management. The decision to use ICIs in BM patients depends on factors such as primary cancer type, metastasis progression, prior treatments, and the patient’s overall health status. Ongoing research is crucial for optimizing therapeutic benefits while minimizing harm. Therefore, biomarker development remains essential to identify patients who will benefit from ICIs versus those at risk of irAEs. Predictive biomarkers like PD-L1, microsatellite instability (MSI), and tumor mutational burden (TMB) are already in clinical use, though challenges in daily practice persist [116]. Emerging biomarkers, such as MMR deficiency and interferon-γ mRNA profiles, show promise in improving irAE predictions and guiding therapy [117,118,119]. Additionally, novel gene signatures like T-cell inflamed gene expression profile (GEP), T-cell dysfunction and exclusion gene signature (TIDE), melanocytic plasticity signature (MPS), and B-cell focused gene signature have shown promise, with MPS offering the best predictive performance [116].

Managing risks requires careful patient selection, close systemic and neurological symptom monitoring, and prompt adverse event management. Moderate to severe irAEs may necessitate treatment pauses and the administration of corticosteroids or immunosuppressive agents such as infliximab or mycophenolate mofetil for steroid-refractory cases [120]. A multidisciplinary approach, especially in managing neurological complications like encephalopathy, is vital to preventing severe toxicity progression [120].

The timing and sequencing of ICI and SRS combinations are critical for BM treatment. Prospective trials are needed to optimize these strategies while developing predictive biomarkers that could revolutionize personalized care. Understanding the long-term impact on neurocognitive function, quality of life, and the pathogenesis of treatment-associated brain necrosis (TABN) will guide more effective management. Evolving clinical guidelines based on ongoing research are necessary to refine combination therapies, minimize toxicity, and identify patient subgroups that will benefit the most from ICIs.

## Figures and Tables

**Table 1:** Summary of clinical trials reporting immune-related adverse events from immune checkpoint inhibitors (ICIs) and combination therapies in brain metastasis (BM) patients.

Trial/Author	Therapy	Patient Cohort	Phase	BM Patients	Grade 3–4 irAEs (and %)
Goldberg et al.	Pembrolizumab + Pemetrexed + Carboplatin	NSCLC with BM	II	42	Pneumonitis (5%), colitis (2%), hypokalemia (2%), adrenal insufficiency (2%), hyperglycemia (2%), and acute kidney injury (2%).
CheckMate 204	Nivolumab + Ipilimumab	Melanoma with BM	II	119	Elevated liver enzymes (15% for asymptomatic); neurologic irAEs (7% for asymptomatic; 17% for symptomatic); and colitis, diarrhea, and hypophysitis (5%)
Kluger et al.	Pembrolizumab	Melanoma with active BM	II	23	Hepatitis (4.3%), hyponatremia (4.3%), and rash (4.3%)
Atezo-Brain (GECP17/05)	Atezolizumab + Carboplatin + Pemetrexed	NSCLC with untreated BM	II	40	Acute kidney injury (5%), pneumonitis (5%), febrile neutropenia (2.5%), and neurologic events (12.5%)
CheckMate 9LA	Nivolumab + Ipilimumab + Chemotherapy	NSCLC with treated BM	III	51	Pneumonitis (3%), hepatitis (4%), rash (4%), and colitis (2%)
KEYNOTE 189	Pembrolizumab + Pemetrexed + Platinum	NSCLC with BM	III	39	Pneumonitis (4%), hepatitis (3%), and nephritis (2%)
IMpower133	Atezolizumab + Carboplatin + Etoposide	ES-SCLC with BM	III	35	Neutropenia (39.9%), anemia (13%), thrombocytopenia (9.8%), and pneumonia (5.2%)
CASPIAN	Durvalumab + Platinum-Etoposide	ES-SCLC with BM	III	28	Neutropenia (15%), anemia (10%), pneumonitis (5%), and infection (5%)
ENHANCE-1	Eribulin + Pembrolizumab	TNBC with BM	Ib/II	18	Neutropenia (26%), fatigue (7%), and peripheral neuropathy (7%)
NIBIT-M2	Ipilimumab + Fotemustine vs. Ipilimumab + Nivolumab	Melanoma with untreated BM	II	76	Elevated liver enzymes (30%), diarrhea (11%), and rash (10%)
ABC	Nivolumab + Ipilimumab	Melanoma with active brain metastases	II	79	Diarrhea/colitis (20%), hepatitis (17%), fatigue (11%), and skin reactions (9%)
KEYNOTE-355	Pembrolizumab + Chemotherapy (nab-paclitaxel; paclitaxel; gemcitabine + carboplatin)	TNBC with BM	III	25	Neutropenia (68%), peripheral neuropathy (15%), pneumonitis (2%), and colitis (2%)
IMpassion130	Atezolizumab + nab-Paclitaxel	TNBC with BM	III	30	Neutropenia (8%), peripheral neuropathy (6%), and fatigue (4%)
